# Spatial scale‐dependent phylogenetic signal in species distributions along geographic and elevation gradients in a mountainous rangeland

**DOI:** 10.1002/ece3.4293

**Published:** 2018-10-05

**Authors:** Maral Pashirzad, Hamid Ejtehadi, Jamil Vaezi, Richard P. Shefferson

**Affiliations:** ^1^ Department of Biology Faculty of Sciences Ferdowsi University of Mashhad Mashhad Iran; ^2^ Organization for Programs on Environmental Sciences, Faculty of Arts & Sciences University of Tokyo Tokyo Japan

**Keywords:** biogeographical processes, community assembly, ecological mechanisms, herbaceous and shrub assemblages, phylogenetic signal

## Abstract

The mechanisms determining community phylogenetic structure range from local ecological mechanisms to broad biogeographical processes. How these community assembly processes determine phylogenetic structure and patterns in rangeland communities across multiple spatial scales is still poorly understood. We sought to determine whether the structure of herbaceous and shrub assemblages along local environmental gradients (elevation) and broad geography (latitude) exhibited phylogenetic signal at different spatial scales, across 2,500 ha of a mountainous rangeland. We analyzed species distribution and phylogenetic data at two spatial scales: the community level (1 m^2^ sample units obtained by stratified random sampling) and the habitat level (plant assemblages identified categorically based on environmental and geographical variables). We found significant phylogenetic signal in structure and pattern at both spatial scales, along local elevational, and latitudinal gradients. Moreover, beta diversity was affected by different environmental variables in herbaceous and shrub species distributions across different spatial scales. Our results highlight the relative importance of local ecological mechanisms, including niche‐based deterministic processes (environmental filtering and species interactions) as well as those of biogeographical processes, such as stochastic dispersal limitation and habitat specialization in plant assemblages of mountainous rangeland.

## 1 INTRODUCTION

The regional plant species pool in rangelands depends on the dispersal ability of species in the greater regional level, and the tolerance of species to abiotic and biotic factors (Willis, 2010). At small spatial scales, rangeland community composition is often considered to result from (a) local abiotic (environmental filtering), (b) local biotic (competition and facilitation interactions), and (c) broad scale processes that sort species from a larger pool (Webb, Ackerly, McPeek, & Donoghue, 2002). However, species interactions generate local‐scale diversity through evolutionary processes, especially in environmentally harsh ecosystems such as rangelands (Benton, 2009). At regional scales, diverse biogeographic processes such as habitat specialization via niche evolution and dispersal limitation may also affect different areas within a region, and different lineages and shape patterns of species diversity and turnover within habitats (Harrison & Grace, 2007). Understanding the primary drivers of biodiversity at different spatial scales and disentangling their relative importance is a fundamental goal of ecology and evolutionary biology (Allan et al., 2011).

Several theories explain the mechanisms shaping local communities. Niche‐based theories posit that deterministic processes such as environmental filtering and biotic interactions affect plant communities, whereas neutral theories suggest stochastic processes, including historical processes and dispersal limitation (Hubbell, 2001; Yang et al., 2015). Dispersal limitation can cause closely related species to occupy close sites. On the one hand, species distributions along environmental gradients depend on both the spatial structure of environmental variables and the tolerance of species to harsh environments such as cold or drought (Pellissier et al. 2013; Qian, Zhang, Zhang, & Wang, 2013). The interactions of species with their environment are mediated by phenotypic traits, which reflect adaptive tradeoffs and may suggest deep or shallower level divergences in species (Jin et al., 2015). Because phylogenetic community structure (PCS) is highly scale and context dependent, the relative importance of niche based and neutral theories differs at different spatial and environmental scales (Cavender‐Bares, Kozak, Fine, & Kembel, 2009; Swenson, Enquist, Pither, Thompson, & Zimmerman, 2006; Kraft et al., 2007; Vamosi et al., 2009).

The relative importance of stochastic and deterministic processes in shaping rangeland plant communities remains particularly unclear (Willis et al., 2010). Phylogenetic beta diversity indices (PBD) are useful means to disentangle the relative importance of these processes during community assembly (Anderson et al., 2011; Kraft et al., 2011). Phylogenetic beta diversity indices can enable us to detect the evolutionary depth of changes (because of their sensitivities to the depth of phylogenetic turnover) and to distinguish different processes in shaping communities at various spatial scales (Graham & Fine, 2008). For example, turnover in both the deep branches and at the tips of a phylogenetic tree in some communities along environmental gradients suggests different processes at work, so that the former can be explained by niche tracking the environment, while the latter can be explained by selection promoting divergence into habitats (Jin et al., 2015). Correlations between PBD and environmental distances may represent adaptive selection resulting from limitations in niche evolution (Eiserhardt et al., 2013), while correlations with spatial distance may reflect dispersal limitation that results in allopatric speciation (Eiserhardt et al., 2013). Investigations integrating both local and regional spatial scales of PCS and phylogenetic turnover have been rare especially on rangeland plant communities (Swenson et al., 2012).

Here, we investigated the relative importance of environment and space on the distribution of herbaceous and shrub species in a mountainous rangeland ecosystem. To obtain a deeper understanding of the processes that influence species distribution along environment and broad geography, we analyzed phylogenetic structure and phylogenetic turnover at different depths of the phylogeny. Because of the high scale dependence of processes shaping plant communities, our study combines two spatial scales: the community and the habitat scales. We analyzed variation partitioning respect to geographical and environment variables to answer: Do space and environment play crucial roles in species assemblage across environmental or geographic gradients? Are certain clades particularly affected by these gradients?

## 2 MATERIAL AND METHODS

### 2.1 Study area

**Figure 1:**
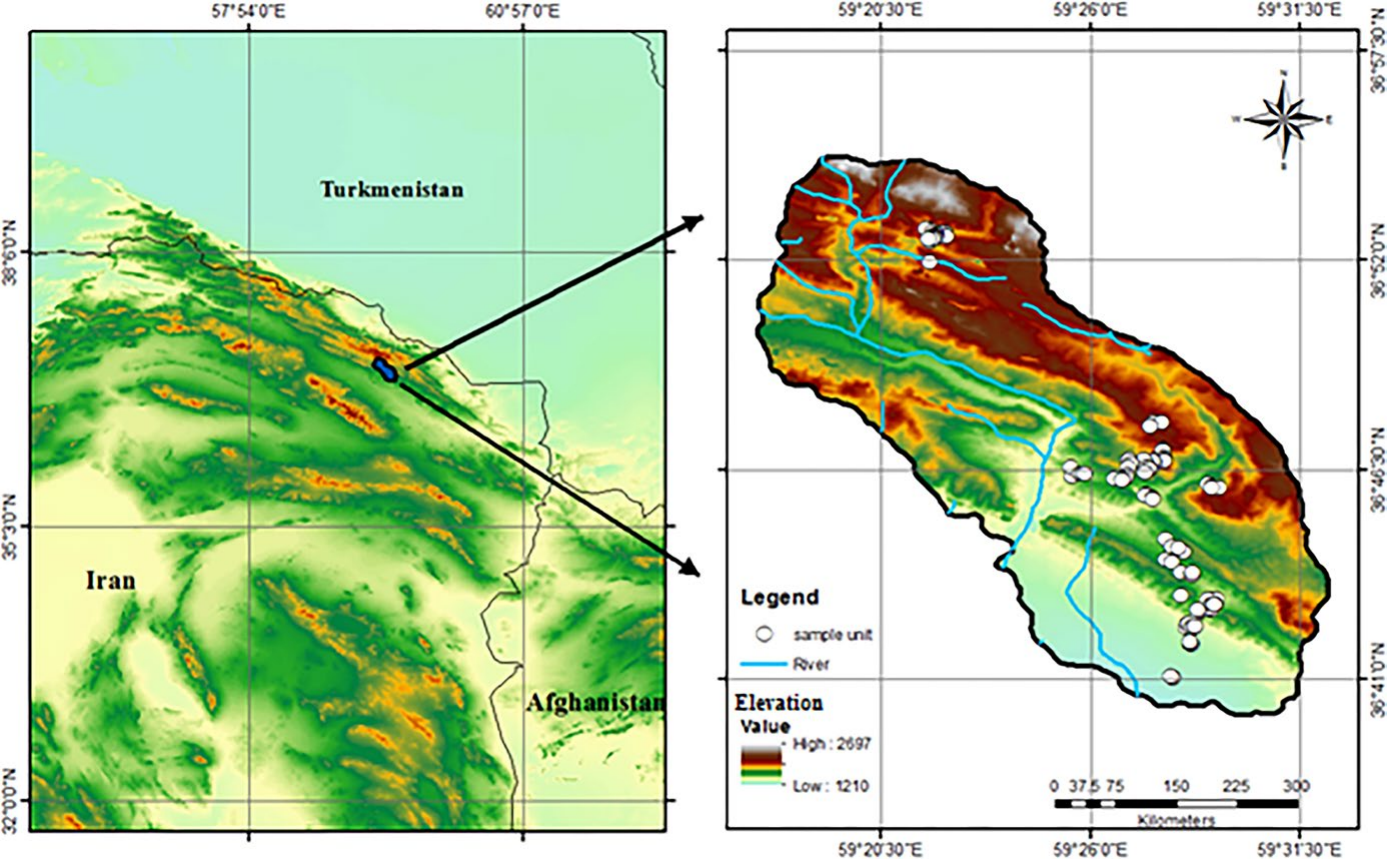
Locations of the 236 sampling units scattered in 2,500 ha from mountain rangelands of south range of Hezar‐Masjed Mountains. Elevation values for sampling unit and the rivers along latitudinal gradient are shown

Our study region is a 2,500‐ha mountainous rangeland in the southern range of the Hezar‐Masjed Mountains between Andad to Amrudak. This area is located in Khorassan Razavi province in northeastern Iran, between 36°40′ and 36°55′N, 59°17′ and 59°31′E. Elevation ranges from 1,200 to 2,200 m and increases while traveling from South to North (Figure 1). Mean annual precipitation ranges from 0 to 45 mm, and mean annual temperature is 6°C. Significant rainfall occurs in the fall and winter, whereas the spring and summer are dry and hot (see Supporting Information Appendix S1). Plant communities are typically dominated by herbaceous plants and shrubs.

### 2.2 Data collection

We set 236 plots (1 m^2^) across elevation (1,200–2,200 m) and latitude (36°40′ and 36°53′N). We divided the study region into four elevation zones (1,200–1,500 m, 1,500–1,800 m, 1,800–2,000 m, and 2,000–2,200 m). Within each zone, we randomly placed 59 plots. To examine the relationship between elevation gradient and climate, we extracted mean annual precipitation and mean annual temperature for all of sample units from the WorldClim v1.4 database (Hijmans et al., 2005). Mean annual temperature decreases about 6°C and mean annual precipitation increases around 60‐mm‐along elevation gradient (see Supporting Information Appendix S2). Latitude was split by geological barriers, most notably rivers (Figure 1). We recorded the identified species and measured their abundance by the number of individuals found. All plant species within the plots were identified, with 168 gymnosperm and angiosperm taxa identified in total (species, subspecies, and varieties), comprising 128 genera across 40 families. Fabaceae, Asteraceae, Brassicaceae, and Poaceae were the four most common families in terms of species richness.

### 2.3 Data analysis

#### 2.3.1 Plant community analysis for determination of herbaceous and shrub assemblages

We defined herbaceous and shrub assemblages on two different scales, the sampling unit (community) level, and the habitat level. Accordingly, we studied plant species composition in 1 m^2^ plots at the community scale.

To identify plant assemblages at the habitat scale along elevation and latitudinal gradients, we used multivariate regression trees (MRT) via the proposed CART extension to handle our response variables (De'ath, 2002). MRT was employed to predict habitat scale herbaceous and shrub assemblages (MRT clusters) from communities (sampling plots). The clusters and their dependence on the environment and geographic variables are presented by a tree. Each cluster indicates a species assemblage on the habitat scale. Moreover, the environmental values define an associated habitat. Our selective tree was based on minimum CVRE (De'ath, 2002; Krishnadas et al. 2016). Accordingly, each1 m^2^ plot was assigned to one of the four habitat types (H1, H2, H3 and H4) in the study region (the spatial distribution of the four habitat types is given in Supporting Information Appendix S3). This analysis was performed by *mvpart* ver.1.3‐1 (De'ath, 2010) package in *R* software version 3.2.5 (R Core Team, 2017).

To identify indicator species that have statistically significant associations with each habitat‐type, we used indicator species analysis (Dufrene & Legendre, 1997). For this, we used habitats identified with untransformed variables from the model with the lowest CVRE. Indicator value (IndVal) is the product of relative abundance and relative frequency of occurrence of the species within a habitat compared to all other habitats. When IndVal equals 1, a species occurs in all plots in a given habitat type, but is absent from other habitats. Statistical significance is determined by multiple randomizations of species occurrences across all plots and comparing observed IndVal with this null distribution (De Cáceres & Legendre, 2009; Dufrene & Legendre, 1997). Species with high IndVal for a habitat are regarded as strong indicators.

#### 2.3.2 Phylogenetic analysis

To estimate phylogenetic diversity and relatedness, we needed a phylogeny that appropriately models the regional species pool (Kress et al. 2009; Kraft & Ackerly, 2014; Lopez‐Angulo, Swenson, & Cavieres, 2018; Qian, Chen, & Zhang, 2017; Qian & Jiang, 2014). We developed our regional species pool as the full list of plant species identified within our sampling plots. This method is the most common in determination of species pool in phylogenetic community ecology (Wang et al., 2013; Webb et al., 2002); however, there are other accepted methods that can be used (De Bello, 2012; Karger et al., 2016). The final list included 168 species plants of gymnosperms and angiosperms. For each plant species, two sequences were used: one plastid DNA gene (rbcL) and one nuclear DNA gene (ITS, containing ITS1, 5.8s and ITS2). Some of the plant sequences were obtained from GenBank and for other species silica‐dried samples, and herbarium leaves were used for DNA extraction using a modified CTAB protocol (Joly et al., 2006). We developed PCR amplification using the standard methodology for Takara ExTaq and sequenced the plant *ITS* and *rbcL* regions for plant that taxons with primers ITS1 and ITS4 (White, Burns, Lee, & Taylor, 1990) and *rbcL*a‐F and *rbcL*a‐R (Kores, Cameron, Molvray, & Chase, 1997), respectively. Plant sequences were assembled using ChromasPro software version 2.1.4 and aligned using MAFFT software (Katoh & Standley, 2013).

#### 2.3.3 Phylogenetic reconstruction

In the field of community ecology, most community studies lack DNA sequence data for the species and taxon‐based phylogenies (Bremer, Bremer & Chase 2009) has been organized with programs, such as Phylomatic (Webb & Donoghue, 2005). Such phylogenetic tree usually only resolved phylogenetic relationships in the ordinal and family levels. Therefore, we conducted additional analyses on DNA sequences for a more thorough exploration of tree topology and branch lengths. We developed our phylogenies as Bayesian reconstructions using MrBayes version 3.2.6 (Ronquist et al., 2012). Parameters were estimated via Markov chain Monte Carlo (MCMC) simulations for one million generations (see Supporting Information Appendix S4a). Second, we generated a maximum‐likelihood phylogeny using PHYML with a BIONJ starting tree (Guindon & Gascuel, 2003; see Supporting Information Appendix S4b). Third, we generated a phylogenetic supertree using the online software Phylomatic (Webb & Donoghue, 2005) that uses the APG III (Chase et al., 2009) topology as the backbone tree onto which taxonomic relationships were grafted (see Supporting Information Appendix S6). The branch lengths of our phylomatic tree were assigned to the tree using the BLADJ algorithm in Phylocom 4.1 (Webb, Ackerly, & Kembel, 2008), and estimation of angiosperm node ages was taken from Wikstrom et al. (2004). For other trees, branch lengths were estimated via the chronos function, which uses the penalized maximum likelihood method to estimate divergence times developed by Sanderson (Sanderson, 2002). When compared, the maximum‐likelihood tree closely matched the topology of the APG III ordinal‐level phylogeny (see Supporting Information Appendix S4b) with lower significant discordances than MrBayes tree among the 22 orders. In some cases where the APG III tree did not resolve ordinal relationships (within the asterids and the rosids), the maximum‐likelihood phylogeny did (Lamiales, Solanales, Boraginales, Malpighiales, Brassicales, Asterales, and Poales). The topology of families within the major groups of angiosperms as defined on the maximum‐likelihood tree was also concordant with the APG III classification (see Supporting Information Appendix S5). Therefore, we only report results based on the maximum‐likelihood molecular phylogeny in the main text.

#### 2.3.4 Community phylogenetic analysis

We estimated the mean pairwise phylogenetic distance (MPD) and mean nearest taxon phylogenetic distance (MNTD) among species in each plot and each habitat to evaluate spatial changes in the phylogenetic structure of community and habitat herbaceous and shrub assemblages (Webb et al., 2002; Swenson 2011). These indices were compared to null models for evaluation of differentiation from random expectations. Random communities were generated by maintaining the species frequency in each plot, but the identities of those species were determined by random draws from the whole species pool. We estimated the standardized MPD and MNTD effect sizes (SES) of MPD and MNTD to describe the “basal” and “terminal” structure of the phylogenetic tree, respectively (Webb et al., 2002). This is important because different processes may act at different evolutionary time scales (Mazel et al., 2016). These analyses were performed using the R package picante (Kembel et al., 2010) in R version 3.2.5. Finally, we used regression analysis to evaluate relations of phylogenetic community structure (SES.mpd and SES.mntd) with elevation and geographic gradients.

#### 2.3.5 Partitioning of phylogenetic beta diversity of herbaceous and shrub assemblages

**Figure 2:**
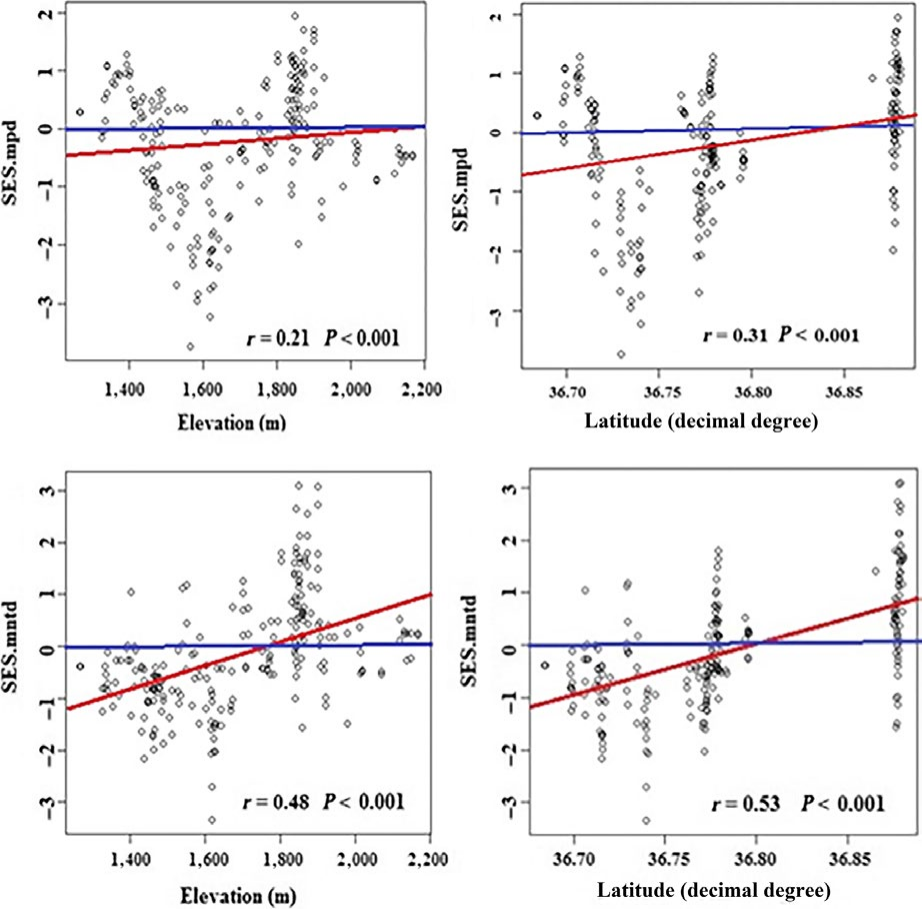
Relations of SES.mpd and SES.mntd with elevation and latitude for community assemblages along elevation and latitude gradients in Hezar‐Masjed regions

Phylogenetic beta diversity was analyzed between plots at the community spatial scale (1 m^2^), and between habitats at the habitat scale (Table 1, Figure 2). We estimated phylogenetic dissimilarity between each pair of plots and habitats using the mean pairwise phylogenetic distance (Dpw) and the mean nearest neighbor phylogenetic distance (Dnn; Cadotte & Davies, 2016; Yang et al., 2015). Dpw and Dnn represent the two main mathematically independent classes (basal and terminal metrics) of phylogenetic metrics, with Dpw sensitive to turnover near the tips of the trees, and Dnn evaluating deeper turnover in the phylogeny (Jin et al., 2015). Lastly, we estimated the standardized effect sizes of Dpw (SES.Dpw) and Dnn (SES.Dnn) using the null distribution. A positive SES.Dpw or SES.Dnn value indicated a higher observed Dpw or Dnn than expected, and a negative SES.Dpw or SES.Dnn value indicated a lower observed Dpw or Dnn than expected.

**Table 1:** The variation of phylogenetic relatedness between herbaceous and shrubs within each habitat explained by elevation and latitude using regression

Phylogenetic distance	Elevation variable	Latitude variable
SES.mpd	0.73^a^	0.86^a^
SES.mntd	0.90^a^	0.75^a^

a *p* < 0.05.

Finally, to evaluate the relative importance of environment and space in shaping rangeland plant communities, we analyzed phylogenetic beta diversity across elevation and geographical distances using Mantel tests and partial Mantel tests. Because habitat‐level herbaceous and shrub assemblage points were independent in the distance matrices, we regressed plant assemblages at the habitat level across explanatory variables of elevation and geographic distance with the *vegan* package (Oksanen et al., 2011) in R version 3.2.5. We tested two hypotheses via partial Mantel tests. First, we identified dispersal limitation occurring when phylogenetic beta diversity was correlated with space independent of environment. Second, we identified environmental filtering as phylogenetic turnover correlating with environment independent of space. Partial mantel tests were used to calculate the correlation between beta and geographical distance given the environment or the correlation between beta and environment given the spatial distance (Cadotte & Davies, 2016).

## 3 RESULTS

### 3.1 Habitat identification and determination of herbaceous and shrub assemblages at the habitat level

We identified four habitat types via multivariate regression tree analysis. The best fit tree in this analysis had a CVRE of 0.56 (*R* ^2^ = 0.43). The first split based on elevation (≥ or <1,799) explained variation (50%) in community composition across all plots. High elevation plots were further segregated based on latitude (> or ≤36.88; See Supporting Information Appendix S7).

Of 168 species, 47 (~34%) showed were significantly associated with only one habitat. The number of species occurring in single habitats ranged from 9 (in habitat H2) to 14 species (habitat H4). In general, high elevation and latitude habitats had more species associated with them (See Supporting Information Appendix S8).

### 3.2 Phylogenetic community structure of herbaceous and shrub assemblages across geographic and elevation gradients

The phylogenetic structure of plant assemblages at both spatial scales was nonrandom with respect to phylogeny along both geographic and environmental gradients (Figure 2, Table 1). Specifically, overdispersion increased significantly across elevation and latitude at the community and habitat scales (*p* < 0.05; Figure 2 and Table 1). Additionally, species occurring together in habitats H1 and H2 tended to be significantly phylogenetically clustered (SES.mpd < 0 or SES.mntd < 0, *p* < 0.05), while herbaceous and shrub assemblages in habitats H3 and H4 tended to be phylogenetically overdispersed (Table 2). Moreover, SES.mpd and SES.mntd were significantly correlated with elevation and latitude in community and habitat scale assemblages, but the standard effect sizes of mntd were more strongly associated with environment and latitude than the standard effect sizes of mpd (Figure 2, Table 1).

**Table 2:** Phylogenetic relatedness between herbaceous and shrubs within each habitat calculated with two different phylogenetic indices (SES.mpd) and (SES.mntd) using regression

Habitat	SES.mpd	SES.mntd
H1	−1.41	−1.97^a^
H2	−1.28	−1.63
H3	1.77	1.96^a^
H4	1.11	2.36^a^

a *p* < 0.05.

### 3.3 Partitioning of phylogenetic beta diversity for assessing of relative importance of stochastic and deterministic processes in shaping of plant communities

**Figure 3:**
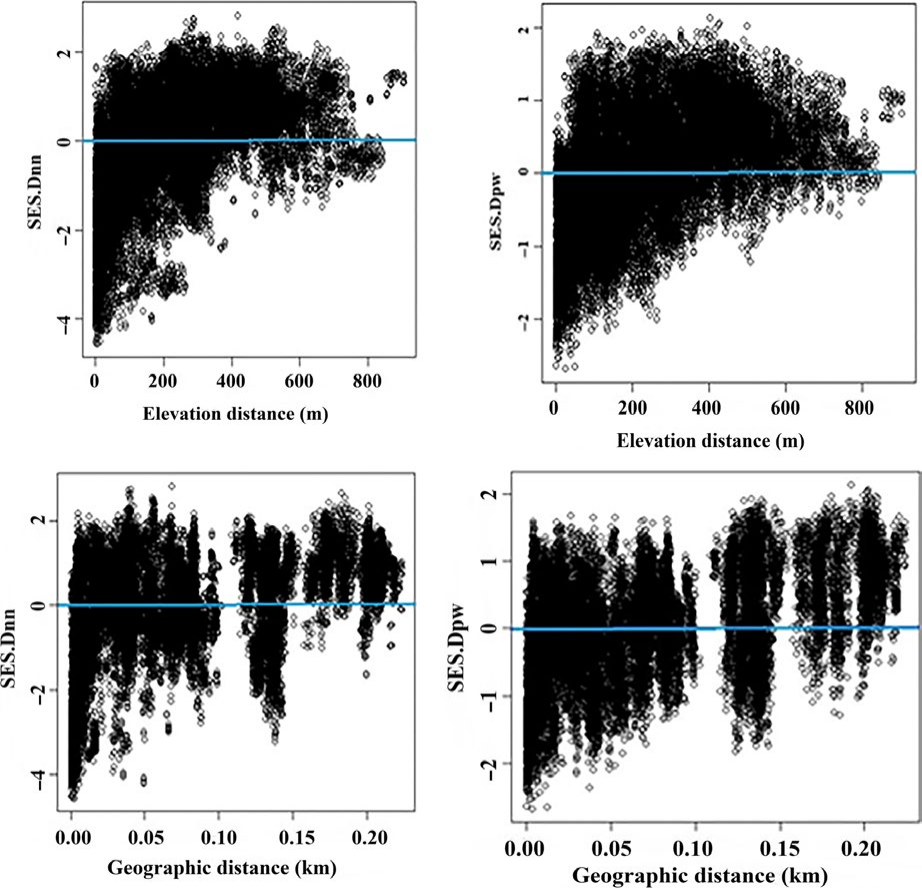
Relationships between elevation distances and geographic distances with standard effect size of phylobetadiversity measures in community plant assemblages (sampling unit). SES.Dnn is terminal metrics of phylogenetic turnover and SES.Dpw is basal metrics of phylobetadiversity

**Figure 4:**
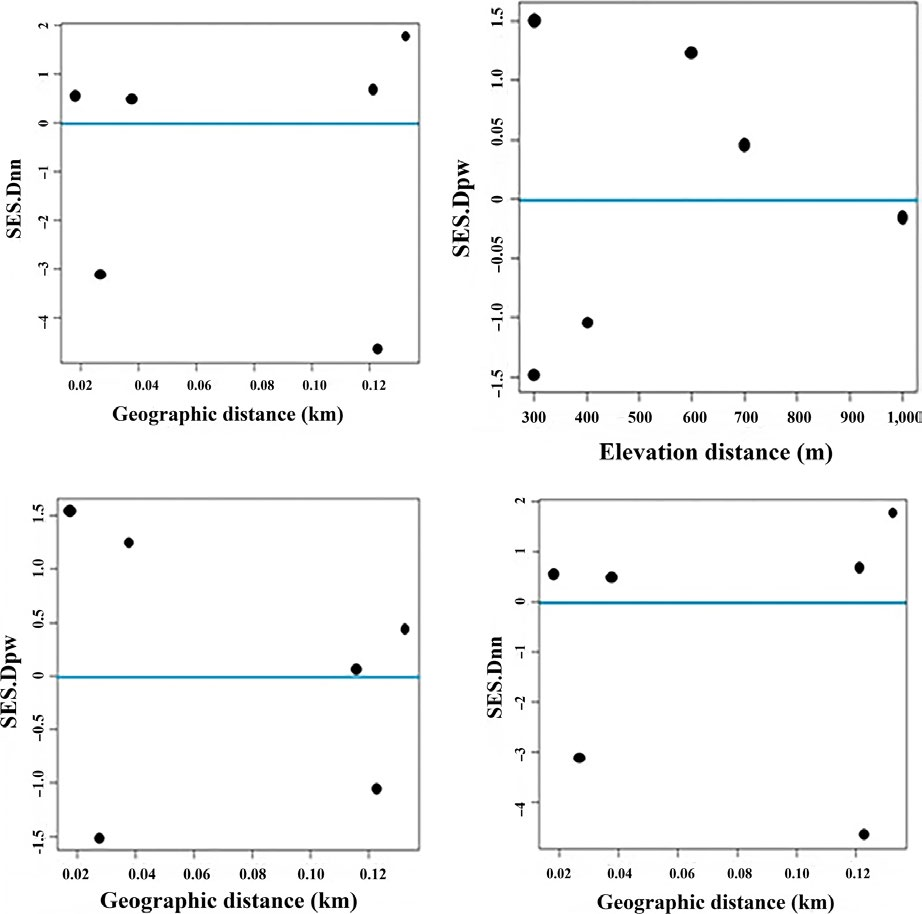
Relationships between geographic distances and elevation distances with standard effect size of phylobetadiversity measures in habitat‐level plant assemblages. SES.Dnn is terminal metrics of phylogenetic beta diversity, and SES.Dpw and is basal metrics of phylobetadiversity

Phylogenetic beta diversity was higher than expected for both community and habitat plant assemblages, as indicated by predominantly positive SES.Dpw and SES.Dnn values (Figures 3 and 4). Phylogenetic dissimilarity between community assemblages moderately increased across elevation, and decreased across geographical distance as the proportion of negative and positive values of phylogenetic dissimilarity indices found along geographical and elevation distances, respectively. This was better explained by terminal metrics, such as the mean nearest taxon phylogenetic distance (Dnn), than by basal metrics such as the mean pairwise phylogenetic distance between assemblages (Dpw; Figure 3, Table 3). Elevation and latitude variables significantly explained phylogenetic beta diversity, but the explanatory power of geographical distance was greater (partial Mantel test, *r* = 0.38, *p* < 0.01) than that of elevation (*r* = 0.22, *p* < 0.01; Table 3). Moreover, Dnn standard effect sizes were more strongly associated with geographical distance than with elevation.

**Table 3:** The variation of phylogenetic dissimilarity explained by geographic and elevation distances for sample unit level plant assemblages using mantel tests and partial mantel tests on distance matrices

Phylogenetic beta diversity index	Elevation distance	Geographic distance	Correlation between beta and geographic distance given elevation	Correlation between beta and elevation distance given geographic variable
*SES. Dpw*	0.35***	0.44***	0.34**	0.20**
*SES. Dnn*	0.38***	0.47**	0.38**	0.22**

***p < 0.001, **p < 0.01, *p < 0.05.

For habitat‐level herbaceous and shrub assemblages, partial Mantel tests reinforced the important effect of elevation (*r* = 0.52, *r* = 0.54 *p* < 0.05) on phylogenetic beta diversity after accounting for the effect of latitude (*r* = 0.37, *r* = 0.30 *p* > 0.05). However, standard effect sizes of Dpw suggested a greater role for elevation in basal phylogenetic beta diversity (Table 4).

**Table 4:** The variation of phylogenetic dissimilarity explained by geographic and elevation distances for habitat level plant assemblages using regression

Phylogenetic beta diversity index	Elevation distance	Geographic distance	Correlation between beta and geographic distance given elevation	Correlation between beta and elevation distance given geographic variable
*SES. Dpw*	0.51^a^	0.20^a^	0.37	0.54^a^
*SES. Dnn*	0.50^a^	0.23^a^	0.30	0.52^a^

a *p* < 0.05.

## 4 DISCUSSION

The phylogenetic structure of rangeland herbaceous and shrub assemblages varied strongly across latitude and elevation. These assemblages tended to consist of distantly related species at higher elevations and latitudes. The observation of phylogenetically more distantly related species in plant assemblages at local and regional scales at high elevation (low temperature) is consistent with predictions that more phylogenetical overdispersion occurs in colder environments due to biotic interactions and environmental filtering (Qian et al., 2017), and with predictions that recourse competition among closely related species drives limited coexistence and niche differences (Machac, Janda, Dunn, & Sanders, 2011). Hence, better association of SES.mntd with studied environment and geography factors than SES.mpd in local scale suggests evolution of cold tolerant species at shallower level (within clades; Qian et al., 2017) and terminal phylogenetic overdispersion in those environments (Mazel et al., 2016). However, in regional scale, stronger correlation of SES.mpd with geography than environment suggests dispersal limitation tolerance evolved in major clades (deep divergences) but positively and stronger correlation of SES.mntd with environment than geography indicated cold tolerant herbaceous and shrub species (cold habitats) evolved more in near to tip of phylogeny (Webb et al., 2002).

Although competitive exclusion as a process has a strong effect on plant communities, facilitation also has an important role in shaping communities especially in environmentally harsher ecosystems such as rangelands (Soliveres et al. 2012; Cavieres et al. 2013; Valiente‐Banuet & Verdu, 2013). In other studies of phylogenetic community structure (PCS) in North and South America, Europe and New Zealand (Butterfield et al., 2013), and the desert and Mediterranean communities of Central America (Valiente‐Banuet & Verdú, 2007), species interactions explained the association of phylogenetically distant species (Butterfield et al., 2013; Iyengar, Bagchi, Barua, Mishra, & Sancaran, 2017). In addition, the facilitative and competitive roles of species have been shown to organize community structure and diversity in rangelands. In our mountainous rangeland, cushion plants may serve as facilitators for community succession in these environments (Brooker et al., 2008). There are many shrub species that increased in density at high elevation or latitude, including cushion plants. These findings indicate significant phylogenetic signal in the structure of herbaceous and shrub assemblages, which can be result from competition among close relatives (Li et al., 2015) and facilitation among distant relatives (Valiente‐Banuet & Verdú, 2007) in colder environments.

Niche‐based deterministic and neutrality‐based stochastic processes were important in our study, as indicated by the strong effects of latitude and elevation on the phylogenetic turnover of herbaceous and shrub species. However, geography was a better predictor of phylogenetic beta diversity at the local scale. Evaluations of terminal and basal phylogenetic turnover metrics with geographic distances indicate there are greater turnover within clades than among clades, even though both are significantly correlated with geographic distances which we infer to mean that the dispersal limitations are relatively conserved near the tips of the phylogeny. Therefore, stochastic assembly and dispersal limitation have more prominent roles in explaining variation at local scale (Gilbert & Lechowicz 2004; Zhang et al., 2013). This may be due to dispersal limitation imposed by geological barriers such as rivers (Li & Sun, 2017).

Although environment and geographic distance had strong associations with terminal and basal phylogenetic beta diversity metrics, we observed greater turnover among clades than within clades at the regional scale. Therefore, we infer environmental requirements are relatively conserved at shallow levels in the phylogeny. Moreover, high beta diversity between habitats indicated nonrandom patterns between habitats types, suggesting the dominance of particular species in each habitat type (Pitman et al., 2001). Habitat conditions may yield greater phylogenetic distance between indicator species in extreme habitats than in less extreme habitats (Pitman, 2001). These results reflected significant habitat specialization and deterministic niche‐based processes such as environmental filtering drove the phylogenetic beta diversity at our habitat scale, resulting in relatively weak biotic interactions at that scale (Jin et al., 2015; Fine & Kembel, 2011). Environmental impacts on local and regional patterns of species assemblages may indicate: (a) dispersal is correlated with topography and (b) multiple environmental factors correlate across space (Qian et al., 2017). These finding are in agreement with results reported in previous studies (de Bello et al., 2013; Dainese et al., 2015; Lopez‐Angulo et al., 2018) that suggest different process acting at different spatial scales shape plant assemblages in mountain regions worldwide.

## 5 CONCLUSION

In conclusion, we found significant phylogenetic signal in the structure and turnover of herbaceous and shrub species distributions of a mountainous rangeland located in the Northeast of Iran. The structure of herbaceous and shrub assemblages at both the community and habitat scales indicated significant phylogenetic overdispersion across elevation and latitude due to niche‐based deterministic processes and species interactions on the local scale and environmental filtering and habitat specialization at the regional scale of herbaceous and shrub assemblages. We particularly noted a different importance to stochastic and deterministic processes on the distribution of species assemblages at different scales. The greater explanatory power of geographic distance and latitude than elevation suggests stochastic processes and dispersal limitation create greater phylogenetic turnover at the tips of branches at the local scale, but a stronger association of terminal PBD with elevation distance at the habitat scale suggests that environmental requirements are conserved shallower in the phylogeny.

## DATA ACCESSIBILITY

Data will be made available in the Dryad Digital Repository.

## CONFLICT OF INTEREST

None declared.

## AUTHOR CONTRIBUTIONS

MP performed the project, wrote the MS and analyzed all of data as the Ph.D. student. HE defined the project as the main supervisor and considered the whole analysis. JV collaborated as the co‐supervisor of the project. RS considered the phylogenetic data analysis and edited the MS as the advisor, also allocated his laboratory in Tokyo University to carry out molecular experiments.
