# Supplementary material for: Spatial scale‐dependent phylogenetic signal in species distributions along geographic and elevation gradients in a mountainous rangeland
Source: Ecol Evol. 2018 Oct 5;8(21):10364–73. doi: 10.1002/ece3.4293 (PMC6238123; doi:10.1002/ece3.4293)
Supplement: Supplementary file 1 [file ECE3-8-10364-s001.docx]

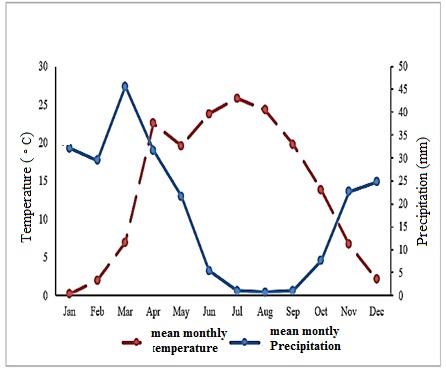


Appendix S1. Emberothermic curve for 2500 ha of a mountainous rangeland in South range of HezarMasjed Mountains located in Northeast of Iran.


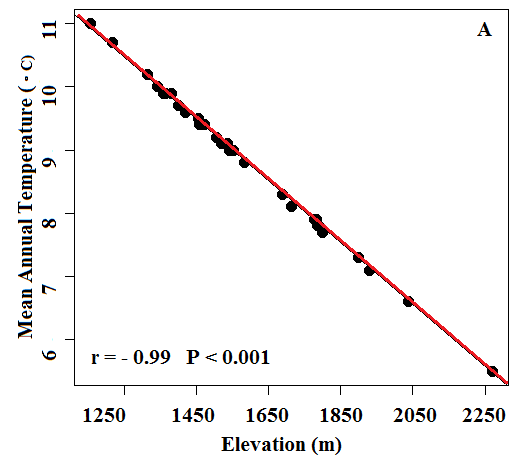

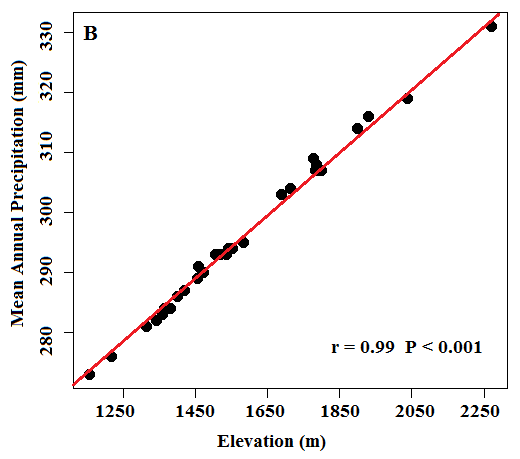


Appendix S2. Relationship between mean annual temperature (A) and mean annual precipitation (B) of studied sites with elevation gradient in studied mountainous semi-arid rangeland.


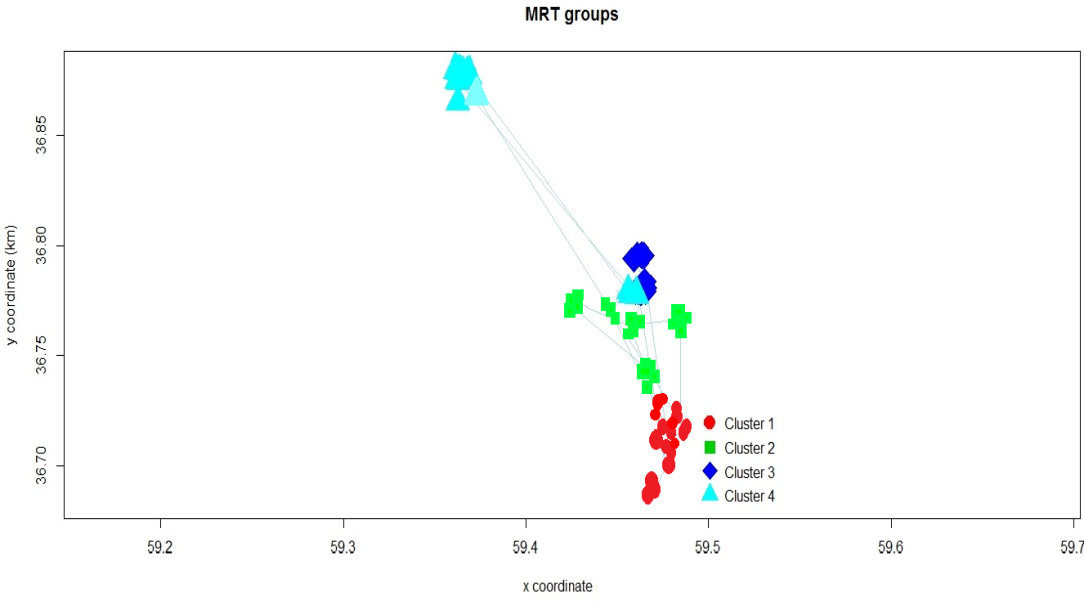

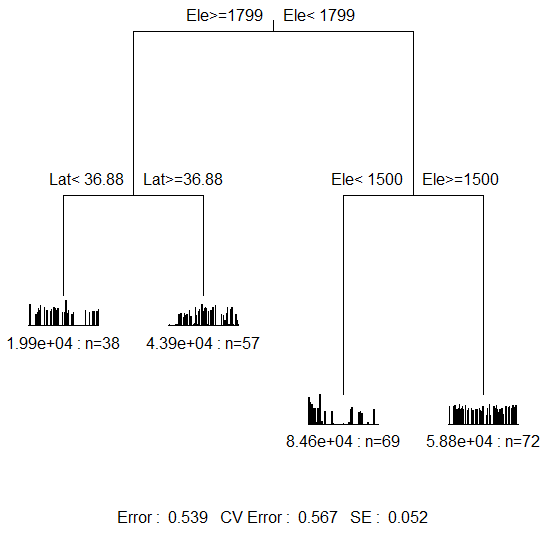


Appendix S3. a. Multivariate regression tree of Hezar-Masjed regions explained by their elevation and latitude variables. b. Clustering with contiguity constraint using MRT.


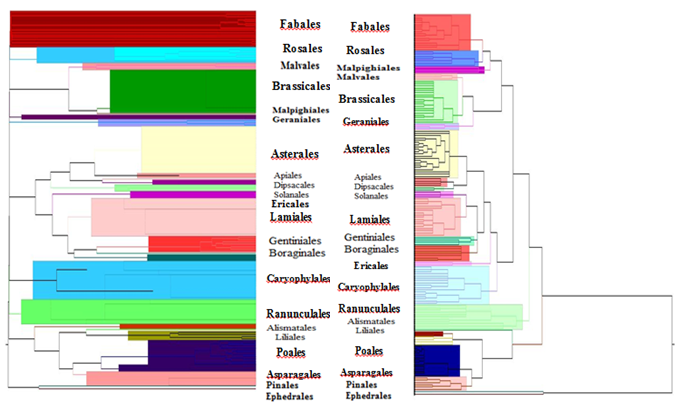

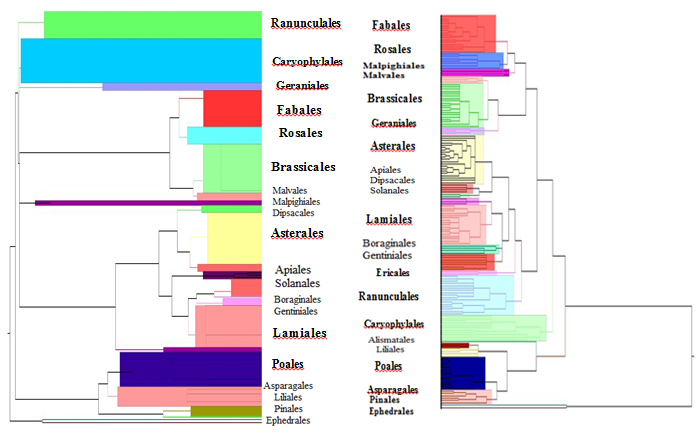


**A**

**B**

Appendix S4. Comparison of phylogenetic relationships based on barcode sequence data versus the Angiosperm Phylogeny Group (APG III). (A) Comparison of the phylogenetic relationships of 22 orders of flowering plants found in Communities and habitats between the Bayesian analysis of the sequence data on the left-hand side and the Angiosperm Phylogeny Group (APG III, 2003) on the right-hand side. (B) Comparison of the phylogenetic relationships of 22 orders of flowering plants found in Communities and habitats between the Maximum likelihood analysis of the sequence data on the left-hand side and the Angiosperm Phylogeny Group (APG III, 2003) on the right -hand side.


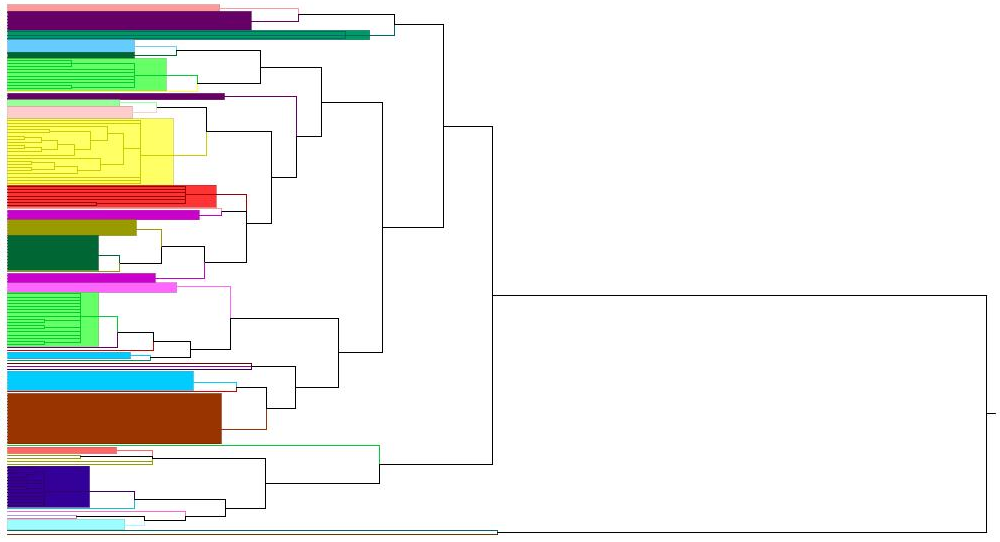

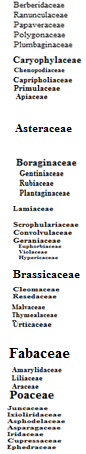

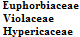

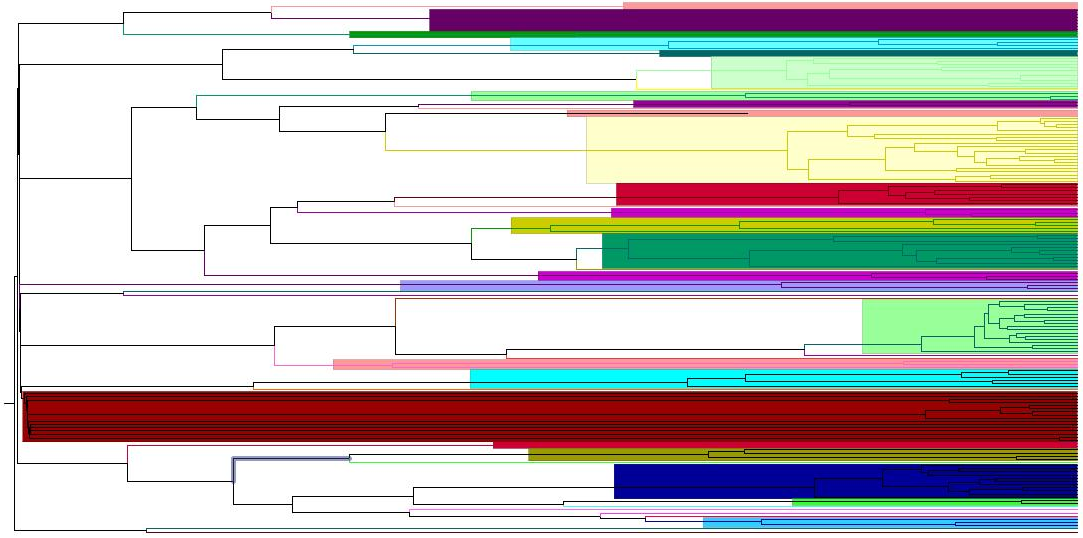


Appendix S5: Comparison of the family-level relationships between the maximum likelihood analysis of the sequence data on the left hand side and the Angiosperm Phylogeny Group (APG III, 2003) on the right hand side.


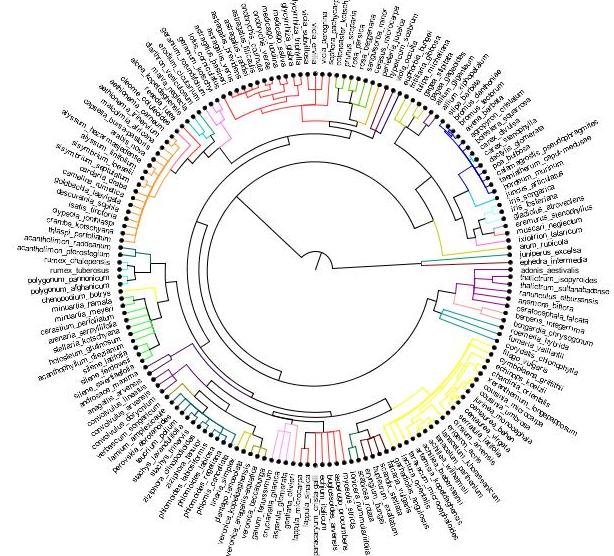


Appendix S6: List of 168 plant species within communities and habitats of a mountainous rangeland located in South range of Hezar- Masjed Mountains. These taxa belong to 166 genera and 40 families.

Appendix S7. Description of habitat types based on MRT splits of community data matrix (site x species abundance) in relation to thresholds of abiotic factors.

| Habitat types | Environmental Characteristics | Number of sample units | Number of plant species |
| --- | --- | --- | --- |
| H1 | Ele<1799,Ele<1500 | 69 | 99 |
| H2 | Ele<1799,Ele>=1500 | 72 | 110 |
| H3 | Ele>=1799,Lat>=36.88 | 57 | 57 |
| H4 | Ele>=1799,Lat<36.88 | 38 | 52 |

The environmental conditions of a habitat are the combination of abiotic variables that were most important in reducing within-group homogeneity of plant community data in relation to all other plots. Ele =Elevation and Lat = Latitude.

**Appendix S8.** Details of species that showed statistically significant associations with each habitat type (P < 0.05). IndVal is the indicator value calculated by randomizing species occurrence in all 236 plots. Species have been arranged in descending order of IndVal magnitude within a habitat. Higher IndVal suggests stronger association with the habitat.

| Habitat | Binomial species | Family | IndVal | P-value |
| --- | --- | --- | --- | --- |
| H1 | *Holosteum glutinosum* | Caryophyllaceae | 0.75 | 0.001 |
| H1 | *Stellaria kotschyana* | Caryophyllaceae | 0.73 | 0.001 |
| H1 | *Androsacae maxima* | Primulaceae | 0.71 | 0.001 |
| H1 | *Minuartia meyeri* | Caryophyllaceae | 0.62 | 0.001 |
| H1 | *Alyssum stapfii* | Brassicaceae | 0.52 | 0.01 |
| H1 | *Bromus tectorum* | Poaceae | 0.50 | 0.01 |
| H1 | *Rosa persica* | Rosaceae | 0.46 | 0.01 |
| H1 | *Iris fosteriana* | Iridaceae | 0.38 | 0.01 |
| H1 | *Poa bolbusa* | Poaceae | 0.37 | 0.01 |
| H1 | *Lotus corniculatus* | Fabaceae | 0.28 | 0.01 |
| H1 | *Sophora pachycarpa* | Fabaceae | 0.20 | 0.02 |
| H2 | *Perovskia abrotanoides* | Lamiaceae | 0.78 | 0.001 |
| H2 | *Lonicera nummulariifolia* | Caprifoliaceae | 0.71 | 0.001 |
| H2 | *Taeniatherum caput-medusae* | Poaceae | 0.70 | 0.001 |
| H2 | *Boissiera squarrosa* | Poaceae | 0.70 | 0.001 |
| H2 | *Diarthron vesiculosum* | Thymelaeaceae | 0.70 | 0.001 |
| H2 | *Artemisia kopetdaghensis* | Asteraceae | 0.52 | 0.01 |
| H2 | *Aethionema carneum* | Brassicaceae | 0.31 | 0.01 |
| H2 | *Stachys lavandulifolia* | Lamiaceae | 0.27 | 0.01 |
| H2 | *Cirsium arvense* | Asteraceae | 0.21 | 0.01 |
| H3 | *Gladiolus atroviolacea* | Iridaceae | 0.65 | 0.001 |
| H3 | *Carex stenophylla* | Poaceae | 0.63 | 0.001 |
| H3 | *Tulipa micheliana* | Liliaceae | 0.59 | 0.001 |
| H3 | *Eremurus stenophyllus* | Asphodelaceae | 0.58 | 0.001 |
| H3 | *Erodium cicutarium* | Geraniaceae | 0.53 | 0.002 |
| H3 | *Crucianella gilanica* | Rubiaceae | 0.51 | 0.01 |
| H3 | *Scabiosa olivieri* | Caprifoliaceae | 0.47 | 0.01 |
| H3 | *Onobrychis verae* | Fabaceae | 0.43 | 0.01 |
| H3 | *Ziziphora clinopodioides* | Lamiaceae | 0.40 | 0.01 |
| H3 | *Minuartia hamata* | Caryophyllaceae | 0.37 | 0.01 |
| H3 | *Myosotis stricta* | Boraginaceae | 0.27 | 0.02 |
| H3 | *Filago vulgaris* | Asteraceae | 0.25 | 0.02 |
| H3 | *Garhadiolus angulosus* | Asteraceae | 0.20 | 0.03 |
| H4 | *Juniperus polycarpos* | Cupressaceae | 0.87 | 0.001 |
| H4 | *Rumex chalepensis* | Polygonaceae | 0.78 | 0.001 |
| H4 | *Rosa beggeriana* | Rosaceae | 0.65 | 0.001 |
| H4 | *Allium giganteum* | Amaryllidaceae | 0.57 | 0.001 |
| H4 | *Bromus dantoniae* | Poaceae | 0.54 | 0.001 |
| H4 | *Lappula sinaica* | Boraginaceae | 0.49 | 0.01 |
| H4 | *Viola occulta* | Violaceae | 0.41 | 0.01 |
| H4 | *Thlaspi perfoliatum* | Brassicaceae | 0.36 | 0.01 |
| H4 | *Falcaria vulgaris* | Apiaceae | 0.31 | 0.01 |
| H4 | *Medicago radiata* | Fabaceae | 0.27 | 0.01 |
| H4 | *Agropyron cristatum* | Poaceae | 0.12 | 0.02 |
| H4 | *Cymbolaena griffithii* | Asteraceae | 0.12 | 0.02 |
| H4 | *Anagallis arvensis* | Primulaceae | 0.10 | 0.03 |
| H4 | *Iris songarica* | Iridaceae | 0.09 | 0.03 |
